# Letter to the editor on a paper by Kaivola et al. (2020): carriership of two copies of *C9orf72* hexanucleotide repeat intermediate-length alleles is not associated with amyotrophic lateral sclerosis or frontotemporal dementia

**DOI:** 10.1186/s40478-022-01438-0

**Published:** 2022-09-21

**Authors:** Sterre C. M. de Boer, Lauren Woolley, Merel O. Mol, Maria Serpente, Lianne M. Reus, Rick van Minkelen, Joke F. A. van Vugt, Federica Sorrentino, Jan H. Veldink, Harro Seelaar, Daniela Galimberti, Fred van Ruissen, Simon Mead, Ekaterina Rogaeva, Yolande A. L. Pijnenburg, Sven J. van der Lee

**Affiliations:** 1 Alzheimer Center Amsterdam, Neurology, Vrije Universiteit Amsterdam, Amsterdam UMC Location VUmc, Amsterdam, The Netherlands; 2 Amsterdam Neuroscience, Neurodegeneration, Amsterdam, The Netherlands; 3 MRC Prion Unit at UCL, Institute of Prion Diseases, UCL, London, UK; 4 Department of Neurology and Alzheimer Center Erasmus MC, Erasmus MC University Medical Center, Rotterdam, The Netherlands; 5 Foundation IRCCS Ca’ Granda, Ospedale Maggiore Policlinico, Milan, Italy; 6 Center for Neurobehavioral Genetics, University of California, Los Angeles, CA USA; 7 Department of Clinical Genetics, Erasmus MC University Medical Center, Rotterdam, The Netherlands; 8 Department of Neurology, University Medical Center Utrecht Brain Center, Utrecht University, Utrecht, The Netherlands; 9 Department of Biomedical, Surgical and Dental Sciences, University of Milan, Milan, Italy; 10 Department of Human Genetics, Amsterdam Reproduction and Development Research Institute, Amsterdam University Medical Center, University of Amsterdam, Amsterdam, The Netherlands; 11 Tanz Centre for Research in Neurodegenerative Diseases, University of Toronto, Toronto, ON Canada; 12 Genomics of Neurodegenerative Diseases and Aging, Human Genetics, Vrije Universiteit Amsterdam, Amsterdam UMC Location VUmc, Amsterdam, The Netherlands

Sir/madam,

Pathological hexanucleotide (G4C2)_n_-repeat expansion in *C9orf72* is the most common genetic cause of amyotrophic lateral sclerosis (ALS), as well as frontotemporal dementia (FTD) and FTD-ALS. Since the discovery of the *C9orf72* repeat expansion as cause for ALS/FTD, there have been several contradicting reports whether intermediate repeat lengths are associated with FTD and/or ALS [1–3]. The definition of intermediate repeat length relies on the lower limit for pathological expansions, which has not been well-established. The most studies are using the initially suggested cutoff of 30 repeats [4, 5]. Recently, Kaivola et al*.* added to the existing literature that carriership of two copies of intermediate-length alleles is a strong risk factor for ALS [6]. Given the prior conflicting evidence, their finding warrants replication and as there is considerable overlap of FTD and ALS, we hypothesized that two copies of the *C9orf72* intermediate-length alleles might also be associated with an increased risk of FTD.

In cohorts independent from Kaivola et al*.,* we studied the association of carriership of two intermediate-length hexanucleotide *C9orf72* repeats with ALS, FTD and a range of other neurodegenerative diseases, including primary progressive aphasia (PPA), corticobasal syndrome (CBS), progressive supranuclear palsy (PSP), Parkinson’s disease (PD) and Alzheimer’s disease (AD). In summary, we did not find evidence for an association of the carriership of two *C9orf72* repeat intermediate-length with any of the neurodegenerative diseases.

We collected data from six different cohorts studying neurodegenerative diseases (total n = 15,021) [7–12]. The *C9orf72* lengths in each cohort were measured using comparable PCR or whole genome sequence methods (Additional file 1: Table S1). We excluded participants with a *C9orf72* repeat expansion (using a ≥ 45 repeats threshold following methods of Kaivola et al. [6], n = 295), with an unknown allele length (n = 21), with an unknown phenotype (n = 28) and the phenotypes vascular dementia, mixed dementia and psychiatric diagnoses (n = 593). The remaining 14,084 participants were included for the analysis. We compared controls (n = 9,497) with five different disease classes: (a) ALS (n = 2,054), (b) FTD (n = 1,016), (c) FTD spectrum (FTD, ALS, PPA = 208, PSP/CBS = 8), (d) PD (n = 315), and (e) AD (n = 986). Statistical power and minimal detectable effect sizes (odds ratio’s) were calculated using the Genetic Association Study (GAS) Power Calculator (e.g. the sample size for power calculations of ALS was 11,551 with a case rate of 17.8%, alpha was 0.05). Expected effect sizes were derived from Kaivola et al. [6]. Disease allele frequencies were derived from our control group (Additional File 1: Table S2, Additional File 1: Table S3). We associated the intermediate-length allele thresholds described by Kaivola et al. [6]: (1) ≥ 7/ ≥ 7 repeats, (2) ≥ 7–16/7–16, and (3) ≥ 7/ ≥ 17–45 units. We fitted separate logistic regression models to study the association of traits with each of the three different intermediate-length threshold groups, adjusting for cohort origin. In addition, we performed analysis within region of origin (North-American, United Kingdom, Northern Europe and Southern Europe) followed by a fixed-effects inverse variance meta-analysis. Statistical analyses were performed using RStudio (version 3.5.2, R Development Core team 2010, rmeta package).

Power analyses showed that our study has ~ 100% power to detect the reported association in ALS in all intermediate-length threshold groups. We found no significant association of ALS, FTD and the FTD spectrum with carriership of two copies of *C9orf72* intermediate-length alleles in all three intermediate-length threshold groups (Table 1). The region of origin analysis (Additional File 1: Table S4) followed by a fixed-effects inverse variance meta-analysis showed similar negative results (Additional File 1: Table S5). We explored the association of AD and PD with carriership of two copies of *C9orf72* intermediate-length alleles. No significant association was found. However, sample size was limited in these groups.

We hypothesized that the true effect is smaller than reported by Kaivola et al*.* Therefore, we calculated the minimum odds ratio that we have 90% power for in our sample. For the ≥ 7/17–45 intermediate-length threshold, our study has 90% power to detect odds larger than 2.12 for ALS and 2.77 for FTD.

**Table 1:** Individuals with two *C9orf72* intermediate-length alleles in ALS, FTD, FTD spectrum, PD and AD patients, and controls after exclusion of expansion carriers

Trait	Shorter/longer allele	Controls with longer alleles (%)	Cases with longer alleles (%)	*p*-value	OR [95% CI]
ALS	< 7/ < 7 vs. = > 7/ = > 7	546 (5.7%)	132 (6.4%)	0.97	0.99 [0.76–1.31]
	< 7/ < 7 vs. 7–16/7–16	500 (5.3%)	121 (5.9%)	0.88	0.98 [0.74–1.30]
	< 7/ < 7 vs. = > 7/ = > 17–45	46 (0.5%)	11 (0.5%)	0.60	1.28 [0.51–3.23]
FTD	< 7/ < 7 vs. = > 7/ = > 7	546 (5.7%)	71 (7%)	0.99	1.00 [0.72–1.39]
	< 7/ < 7 vs. 7–16/7–16	500 (5.3%)	64 (6.3%)	0.96	0.99 [0.70–1.40]
	< 7/ < 7 vs. = > 7/ = > 17–45	46 (0.5%)	7 (0.7%)	0.86	1.09 [0.40–3.00]
FTD spectrum	< 7/ < 7 vs. = > 7/ = > 7	546 (5.7%)	217 (6.6%)	0.86	0.98 [0.79–1.22]
	< 7/ < 7 vs. 7–16/7–16	500 (5.3%)	199 (6.1%)	0.81	0.97 [0.78–1.22]
	< 7/ < 7 vs. = > 7/ = > 17–45	46 (0.5%)	18 (0.5%)	0.90	1.05 [0.50–2.20]
PD	< 7/ < 7 vs. = > 7/ = > 7	546 (5.7%)	22 (7%)	0.94	1.02 [0.59–1.76]
	< 7/ < 7 vs. 7–16/7–16	500 (5.3%)	21 (6.7%)	0.73	1.10 [0.63–1.94]
	< 7/ < 7 vs. = > 7/ = > 17–45	46 (0.5%)	1 (0.3%)	0.41	0.41 [0.05–3.50]
AD	< 7/ < 7 vs. = > 7/ = > 7	546 (5.7%)	67 (6.8%)	0.48	0.88 [0.60–1.27]
	< 7/ < 7 vs. 7–16/7–16	500 (5.3%)	60 (6.1%)	0.45	0.86 [0.58–1.27]
	< 7/ < 7 vs. = > 7/ = > 17–45	46 (0.5%)	7 (0.7%)	0.99	1.00 [0.33–3.02]

*ALS* Amyotrophic lateral sclerosis, *FTD* Frontotemporal dementia, *FTD spectrum includes* bvFTD, primary progressive aphasia, corticobasal degeneration and progressive supra nuclear palsy, *PD* Parkinson’s disease, *AD* Alzheimer’s disease *OR* Odds ratio, *CI* Confidence interval, *N.A* not applicable

Several suggestions may explain the discrepancy between Kaivola’s strong positive findings and our negative results. First, the higher prevalence of the intermediate-length alleles in Finland [13] versus the non-Finnish Europeans and North Americans represented in our cohort, could have resulted in the Finnish study to have increased power. Second, there could be another, Finland-specific, pathological variant present on the haplotype with the intermediate length allele that associates with ALS. Likewise, there are sub-haplotypes with an increased ‘base’ repeat-length, predisposing to pathological repeat expansions [10]. Third, the genotyping in the Finnish study and in our study, was not done at one site. This may have resulted in batch or laboratory effects. In our study, we corrected for batch or laboratory effects by adjusting for cohort of origin in our logistic regression models and observed no effects. Still, we cannot fully rule out false negative findings due to cohort or technical biases. In support of the association, a Belgian study showed that lengths of ≥ 7–24 are almost exclusively present on the chromosome 9 risk haplotype tagged by the rs2814707 T-allele and that homozygous carriership of the T-allele is associated with disease (OR = 1.8, *p* = 0.04) [2]. Homozygous carriership of this T-allele was associated with ALS and FTD-ALS (OR = 2.08, *p* = 0.04) in the non-expansion group [1].

We also made an interesting observation when reviewing the clinical records of carriers of two copies of repeat intermediate-length in one cohort. These patients showed a bvFTD phenotype with noteworthy co-symptoms of PSP and ALS. A co-existence that, based on clinicopathology, is not to be expected and as far as we are aware, has not previously been associated with *C9orf72* repeat intermediate-length [14].

Altogether, in this multinational cohort we could not confirm an association of carriership of two copies of *C9orf72* repeat intermediate-length alleles with ALS or FTD.

## Supplementary Information

**Additional file 1.**

**Additional file 2.**
