# Supplementary material for: Letter to the editor on a paper by Kaivola et al. (2020): carriership of two copies of C9orf72 hexanucleotide repeat intermediate-length alleles is not associated with amyotrophic lateral sclerosis or frontotemporal dementia
Source: Acta Neuropathol Commun. 2022 Sep 21;10:141. doi: 10.1186/s40478-022-01438-0 (PMC9494883; doi:10.1186/s40478-022-01438-0)
Supplement: Supplementary file 1 — Additional file 1. [file 40478_2022_1438_MOESM1_ESM.docx]

**Supplementary material – Letter to the Editor – de Boer et al., 2022**

| **Cohort** | **Method length *C9orf72*** |
| --- | --- |
| Beck et al., 2013 | RP-PCR following Renton et al., 2011. Expansions with a characteristic “saw-tooth” pattern were identified and put forward for Southern blotting where sufficient DNA allowed. |
| Huisman et al., 2011 | ExpansionHunter was applied on the G_4_C_2_ repeat using short-read whole genome sequencing data (HiSeq2000 en HiSeqX, 30x minimal coverage). Expansionhunter uses the depth of coverage compared to the average coverage to estimate the length of the expansion ([https://github.com/Illumina/ExpansionHunter](about:blank)). |
| Kaivola et al., 2020 | Renton et al., 2011, with minor modifications, see table S1.B. |
| Mol et al., 2021 | The presence of a *C9orf72* repeat expansion was tested using either repeat-primed PCR or a commercial kit (AmplideX PCR/CE, Asuragen) |
| Reus et al., 2021* | Renton et al., 2011, with minor modifications. Expansions, or if only 1 allel was present, were followed by either repeat-primed PCR or a commercial (AmplideX PCR/CE C9orf72 Kit, Asuragen) kit. |
| Serpente et al., 2021 | The genomic DNA was amplified using a three-primer G4C2-Repeat Primed (RP)-PCR configuration, followed by fragment sizing on a 3100 Genetic Analyzer (Thermo Fisher). ROX 1000 was used for sizing by capillary electrophoresis and the size of the PCR products were converted to the number of G_4_C_2_ repeats using size and mobility conversion factor with GeneMapper v 4.1 software (Thermo Fisher). |
| Xi et al., 2012 | DeJesus-Hernandez et al., 2011. |

**Table S1.A.** Methods of measuring the *C9orf72* repeat lengths per cohort.

*All samples that were genotyped were published before with the exception of samples genotyped after our previous publication (Reus *et al.,* 2021). Examples of the raw PCR data of these samples are shown in the supplementary PDF file entitled “Supplementary_deBoer_rawPCRdata.”

| **Renton et al., 2011:** | **Kaivola et al., 2020:** |
| --- | --- |
| Repeat-primed PCR was performed as follows: | Repeat-primed PCR was performed as follows: |
| 100 ng of genomic DNA were used as template | Reactions containing 50-100ng of genomic DNA |
| Final volume of 28 ul | 10µl final volume |
| 14 ul of FastStart PCR Master Mix (Roche Applied Science, Indianapolis, IN, USA) | 4.7 µl of FastStart PCR master mix (Roche Applied Science, Indianapolis, IN, USA) |
| Final concentration of 0.18 mM 7-deaza-dGTP (New England Biolabs, Ipswich, MA, USA) | Not reported |
| 1x Q-Solution (QIAGEN, Valencia, CA, USA), | 1x Q solution (QIAGEN, Valencia, CA, USA) |
| 7% DMSO (Sigma[1]Aldrich) | Final concentration of 7% DMSO (Sigma-Aldrich, St. Louis, USA) |
| 0.9 mM MgCl2 (QIAGEN) | 0.93 mM MgCl2 (QIAGEN) |
| 0.7  µM reverse primer consisting of four GGGGCC repeats with an anchor tail, | 0.23 µM reverse primer |
| 1.4  µM 6FAM-fluorescent labeled forward primer located 280 bp telomeric to the repeat sequence, and 1.4  µM anchor primer corresponding to the anchor tail of the reverse primer | 0.47 µM of [6FAM] forward primer anchor primer (Sigma-Aldrich), |
| Forward primer 5’->3’  [6-FAM]-AGTCGCTAGAGGCGAAAGC | Forward primer 5’->3’   [6FAM]AGTCGCTAGAGGCGAAAGC, |
| Reverse primer 5’->3’  TACGCATCCCAGTTTGAGACGGGGGCCGGGGCCGGGGCCGGGG | Reverse primer 5’->3’ TACGCATCCCAGTTTGAGACGGGGGCCGGGGCCGGGGCCGGGG and |
| Anchor primer 5’->3’ TACGCATCCCAGTTTGAGACG | Anchoring primer 5’->3’ TACGCATCCCAGTTTGAGAG |
| program was used where the annealing temperature was gradually lowered from 70 C to 56 C in 2 C increments with a 3 min extension time for each cycle. | Identical |
|  | Additional PCR over the repeat to confirm the expansions, which resembles the GC-Rich PCR where we determine if we have 1 or 2 alleles. In conditions with one allele the samples should be confirmed by a repeat primed PCR. |

**Table S1.B.** Comparison of methods measuring *C9orf72* repeat length between Renton et al., 2011 and Kaivola et al., 2020.

| **Trait** | **Allele lengths** | **Cohort** | **Controls** | **%** | **Cases** | **%** |
| --- | --- | --- | --- | --- | --- | --- |
| ALS | (G_4_C_2_)_7-45_/(G_4_C_2_)_7-45_ | **Total** | **546/9497** | **5,7** | **132/2054** | **6,4** |
|  |  | Huisman et al., 2011 | 67/1040 | 6,4 | 102/1681 | 6,1 |
|  |  | Reus et al., 2021 (Amsterdam Dementia Cohort) | 26/274 | 9,5 | n.a. | n.a. |
|  |  | Xi et al., 2012 | 42/602 | 7,0 | 29/370 | 7,8 |
|  |  | Serpente et al., 2021 | 3/14 | 21,4 | 1/2 | 50,0 |
|  |  | Beck et al., 2013 | 408/7565 | 5,4 | n.a. | n.a. |
|  |  | Mol et al., 2021 | 0/2 | 0,0 | n.a. | n.a. |
|  | (G_4_C_2_)_7-16_/(G_4_C_2_)_7-16_ | **Total** | **500/9497** | **5,3** | **121/2054** | **5,9** |
|  |  | Huisman et al., 2011 | 63/1040 | 6,1 | 96/1681 | 5,7 |
|  |  | Reus et al., 2021 (Amsterdam Dementia Cohort) | 24/274 | 8,8 | n.a. | n.a. |
|  |  | Xi et al., 2012 | 37/602 | 6,1 | 25/370 | 6,8 |
|  |  | Serpente et al., 2021 | 3/14 | 21,4 | 0/2 | 0,0 |
|  |  | Beck et al., 2013 | 373/7565 | 4,9 | n.a. | n.a. |
|  |  | Mol et al., 2021 | 0/2 | 0,0 | n.a. | n.a. |
|  | (G_4_C_2_)_7-45_/(G_4_C_2_)_17-45_ | **Total** | **46/9497** | **0,5** | **11/2054** | **0,5** |
|  |  | Huisman et al., 2011 | 4/1040 | 0,4 | 6/1681 | 0,4 |
|  |  | Reus et al., 2021 (Amsterdam Dementia Cohort) | 2/274 | 0,7 | n.a. | n.a. |
|  |  | Xi et al., 2012 | 5/602 | 0,8 | 4/370 | 1,1 |
|  |  | Serpente et al., 2021 | 0/14 | 0,0 | 1/2 | 50,0 |
|  |  | Beck et al., 2013 | 35/7565 | 0,5 | n.a. | n.a. |
|  |  | Mol et al., 2021 | 0/2 | 0,0 | n.a. | n.a. |
|  |  |  |  |  |  |  |

**Table S2.** Synopsis of the *C9orf72* intermediate lengths in ALS per included cohort.

| **Trait** | **Allele lengths** | **Cohort** | **Controls** | **%** | **Cases** | | **%** |
| --- | --- | --- | --- | --- | --- | --- | --- |
| FTD | (G_4_C_2_)_7-45_/(G_4_C_2_)_7-45_ | **Total** | **546/9497** | **5,7** | | **71/1016** | **7,0** |
|  |  | Huisman et al., 2011 | 67/1040 | 6,4 | | n.a. | n.a. |
|  |  | Reus et al., 2021 | 26/274 | 9,5 | | 21/294 | 7,1 |
|  |  | Xi et al., 2012 | 42/602 | 7,0 | | 25/367 | 6,8 |
|  |  | Serpente et al., 2021 | 3/14 | 21,4 | | 2/38 | 5,3 |
|  |  | Beck et al., 2013 | 408/7565 | 5,4 | | 16/232 | 6,9 |
|  |  | Mol et al., 2021 | 0/2 | 0,0 | | 7/86 | 8,1 |
|  | (G_4_C_2_)_7-16_/(G_4_C_2_)_7-16_ | **Total** | **500/9497** | **5,3** | | **64/1016** | **6,3** |
|  |  | Huisman et al., 2011 | 63/1040 | 6,1 | | n.a. | n.a. |
|  |  | Reus et al., 2021 | 24/274 | 8,8 | | 18/294 | 6,1 |
|  |  | Xi et al., 2012 | 37/602 | 6,1 | | 24/367 | 6,5 |
|  |  | Serpente et al., 2021 | 3/14 | 21,4 | | 1/38 | 2,6 |
|  |  | Beck et al., 2013 | 373/7565 | 4,9 | | 14/232 | 6,0 |
|  |  | Mol et al., 2021 | 0/2 | 0,0 | | 7/86 | 8,1 |
|  | (G_4_C_2_)_7-45_/(G_4_C_2_)_17-45_ | **Total** | **46/9497** | **0,5** | | **7/1016** | **0,7** |
|  |  | Huisman et al., 2011 | 4/1040 | 0,4 | | n.a. | n.a. |
|  |  | Reus et al., 2021 | 2/274 | 0,7 | | 3/294 | 1,0 |
|  |  | Xi et al., 2012 | 5/602 | 0,8 | | 1/367 | 0,3 |
|  |  | Serpente et al., 2021 | 0/14 | 0,0 | | 1/38 | 2,6 |
|  |  | Beck et al., 2013 | 35/7565 | 0,5 | | 2/232 | 0,9 |
|  |  | Mol et al., 2021 | 0/2 | 0,0 | | 0/86 | 0,0 |

**Table S3.** Synopsis of the *C9orf72* intermediate lengths in FTD per included cohort.

| **Trait** | **Country** | **Shorter/longer allele** | **Controls with longer alleles (%)** | **Cases with longer alleles (%)** | **p-value** | **OR [95% CI]** |
| --- | --- | --- | --- | --- | --- | --- |
| ALS | North-America | <7/<7 vs. =>7/=>7 | 8 (5.4%) | 19 (8.1%) | 0.3 | 1.59 [0.67-3.78] |
|  | North-America | <7/<7 vs. 7-16/7-16 | 7 (4.7%) | 17 (7.3%) | 0.3 | 1.62 [0.65-4.08] |
|  | North-America | <7/<7 vs. =>7/=>17-45 | 1 (0.7%) | 2 (0.9%) | 0.81 | 1.34 [0.12-14.98] |
|  | Northern Europe | <7/<7 vs. =>7/=>7 | 93 (7.1%) | 102 (6.1%) | 0.22 | 0.83 [0.62-1.12] |
|  | Northern Europe | <7/<7 vs. 7-16/7-16 | 87 (6.6%) | 96 (5.7%) | 0.25 | 0.84 [0.62-1.13] |
|  | Northern Europe | <7/<7 vs. =>7/=>17-45 | 6 (0.5%) | 6 (0.4%) | 0.63 | 0.76 [0.24-2.36] |
|  | Southern Europe | <7/<7 vs. =>7/=>7 | 35 (7.8%) | 3 (11.1%) | 0.47 | 1.60 [0.44-5.86] |
|  | Southern Europe | <7/<7 vs. 7-16/7-16 | 31 (6.9%) | 2 (7.4%) | 0.81 | 1.21 [0.26-5.56] |
|  | Southern Europe | <7/<7 vs. =>7/=>17-45 | 4 (0.9%) | 1 (3.7%) | 0.18 | 4.68 [0.49-44.67] |
|  | United Kingdom | <7/<7 vs. =>7/=>7 | 408 (5.4%) | 7 (7.2%) | 0.26 | 1.58 [0.71-3.52] |
|  | United Kingdom | <7/<7 vs. 7-16/7-16 | 373 (4.9%) | 5 (5.2%) | 0.65 | 1.24 [0.49-3.12] |
|  | United Kingdom | <7/<7 vs. =>7/=>17-45 | 35 (0.5%) | 2 (2.1%) | 0.03 | 5.27[1.23-22.53] |
| FTD | North-America | <7/<7 vs. =>7/=>7 | 8 (5.4%) | 7 (7.5%) | 0.36 | 1.66 [0.57-4.85] |
|  | North-America | <7/<7 vs. 7-16/7-16 | 7 (4.7%) | 6 (6.5%) | 0.41 | 1.62 [0.52-5.11] |
|  | North-America | <7/<7 vs. =>7/=>17-45 | 1 (0.7%) | 1 (1.1%) | 0.65 | 1.90 [0.12-30.98] |
|  | Northern Europe | <7/<7 vs. =>7/=>7 | 93 (7.1%) | 28 (7.4%) | 0.93 | 1.02 [0.65-1.60] |
|  | Northern Europe | <7/<7 vs. 7-16/7-16 | 87 (6.6%) | 25 (6.6%) | 0.91 | 0.97 [0.61-1.56] |
|  | Northern Europe | <7/<7 vs. =>7/=>17-45 | 6 (0.5%) | 3 (0.8%) | 0.46 | 1.70 [0.42-6.84] |
|  | Southern Europe | <7/<7 vs. =>7/=>7 | 35 (7.8%) | 20 (6.4%) | 0.67 | 0.88 [0.49-1.58] |
|  | Southern Europe | <7/<7 vs. 7-16/7-16 | 31 (6.9%) | 19 (6.1%) | 0.85 | 0.94 [0.52-1.73] |
|  | Southern Europe | <7/<7 vs. =>7/=>17-45 | 4 (0.9%) | 1 (0.3%) | 0.4 | 0.39 [0.04-3.48] |
|  | United Kingdom | <7/<7 vs. =>7/=>7 | 408 (5.4%) | 16 (6.9%) | 0.23 | 1.39 [0.82-2.36] |
|  | United Kingdom | <7/<7 vs. 7-16/7-16 | 373 (4.9%) | 14 (6%) | 0.32 | 1.33 [0.76-2.33] |
|  | United Kingdom | <7/<7 vs. =>7/=>17-45 | 35 (0.5%) | 2 (0.9%) | 0.34 | 2.02 [0.48-8.50] |
| FTD spectrum | North-America | <7/<7 vs. =>7/=>7 | 8 (5.4%) | 26 (7.2%) | 0.42 | 1.41 [0.61-3.23] |
|  | North-America | <7/<7 vs. 7-16/7-16 | 7 (4.7%) | 23 (6.4%) | 0.43 | 1.42 [0.59-3.44] |
|  | North-America | <7/<7 vs. =>7/=>17-45 | 1 (0.7%) | 3 (0.8%) | 0.82 | 1.30 [0.13-12.67] |
|  | Northern Europe | <7/<7 vs. =>7/=>7 | 93 (7.1%) | 134 (6.3%) | 0.31 | 0.86 [0.65-1.14] |
|  | Northern Europe | <7/<7 vs. 7-16/7-16 | 87 (6.6%) | 125 (5.9%) | 0.31 | 0.86 [0.65-1.15] |
|  | Northern Europe | <7/<7 vs. =>7/=>17-45 | 6 (0.5%) | 9 (0.4%) | 0.84 | 0.90 [0.32-2.54] |
|  | Southern Europe | <7/<7 vs. =>7/=>7 | 35 (7.8%) | 33 (7.4%) | 0.94 | 1.02 [0.62-1.69] |
|  | Southern Europe | <7/<7 vs. 7-16/7-16 | 31 (6.9%) | 31 (6.9%) | 0.77 | 1.08 [0.64-1.83] |
|  | Southern Europe | <7/<7 vs. =>7/=>17-45 | 4 (0.9%) | 2 (0.4%) | 0.48 | 0.54 [0.10-2.98] |
|  | United Kingdom | <7/<7 vs. =>7/=>7 | 408 (5.4%) | 23 (7%) | 0.11 | 1.44 [0.92-2.25] |
|  | United Kingdom | <7/<7 vs. 7-16/7-16 | 373 (4.9%) | 19 (5.8%) | 0.29 | 1.30 [0.80-2.12] |
|  | United Kingdom | <7/<7 vs. =>7/=>17-45 | 35 (0.5%) | 4 (1.2%) | 0.04 | 2.92 [1.03-8.32] |
| PD | North-America | <7/<7 vs. =>7/=>7 | 8 (5.4%) | 22 (7.1%) | 0.42 | 1.42 [0.61-3.32] |
|  | North-America | <7/<7 vs. 7-16/7-16 | 7 (4.7%) | 21 (6.8%) | 0.33 | 1.55 [0.64-3.79] |
|  | North-America | <7/<7 vs. =>7/=>17-45 | 1 (0.7%) | 1 (0.3%) | 0.64 | 0.52 [0.03-8.36] |
|  | Northern Europe | <7/<7 vs. =>7/=>7 | 93 (7.1%) | 0 (0%) | n.a. | n.a. |
|  | Northern Europe | <7/<7 vs. 7-16/7-16 | 87 (6.6%) | 0 (0%) | n.a. | n.a. |
|  | Northern Europe | <7/<7 vs. =>7/=>17-45 | 6 (0.5%) | 0 (0%) | n.a. | n.a. |
|  | Southern Europe | <7/<7 vs. =>7/=>7 | 35 (7.8%) | 0 (0%) | n.a. | n.a. |
|  | Southern Europe | <7/<7 vs. 7-16/7-16 | 31 (6.9%) | 0 (0%) | n.a. | n.a. |
|  | Southern Europe | <7/<7 vs. =>7/=>17-45 | 4 (0.9%) | 0 (0%) | n.a. | n.a. |
| AD | North-America | <7/<7 vs. =>7/=>7 | 8 (5.4%) | 20 (8.9%) | 0.18 | 1.81 [0.76-4.28] |
|  | North-America | <7/<7 vs. 7-16/7-16 | 7 (4.7%) | 17 (7.6%) | 0.23 | 1.75 [0.70-4.40] |
|  | North-America | <7/<7 vs. =>7/=>17-45 | 1 (0.7%) | 3 (1.3%) | 0.51 | 2.17 [0.22-21.17] |
|  | Northern Europe | <7/<7 vs. =>7/=>7 | 93 (7.1%) | 26 (5.2%) | 0.25 | 0.76 [0.48-1.20] |
|  | Northern Europe | <7/<7 vs. 7-16/7-16 | 87 (6.6%) | 24 (4.8%) | 0.24 | 0.75 [0.47-1.21] |
|  | Northern Europe | <7/<7 vs. =>7/=>17-45 | 6 (0.5%) | 2 (0.4%) | 0.91 | 0.91 [0.18-4.54] |
|  | Southern Europe | <7/<7 vs. =>7/=>7 | 35 (7.8%) | 9 (6.4%) | 0.93 | 1.04 [0.47-2.26] |
|  | Southern Europe | <7/<7 vs. 7-16/7-16 | 31 (6.9%) | 7 (5%) | 0.83 | 0.91 [0.38-2.16] |
|  | Southern Europe | <7/<7 vs. =>7/=>17-45 | 4 (0.9%) | 2 (1.4%) | 0.42 | 2.02 [0.36-11.24] |

**Table S4.** Individuals per region of origin (North-American, United Kingdom, Northern Europe and Southern Europe) with two C9orf72 intermediate-length alleles in ALS, FTD, FTD spectrum, PD and AD patients, and controls after exclusion of expansion carriers.

Abbreviations: ALS = amyotrophic lateral sclerosis, FTD = frontotemporal dementia, FTD spectrum = bvFTD, primary progressive aphasia, corticobasal degeneration (CBD) and progressive supra nuclear palsy (PSP), PD = Parkinson’s disease, AD = Alzheimer’s disease. OR = odds ratio, CI = confidence interval, n.a. = not applicable.

| **Trait** | **Shorter/longer allele** | **p-value** | **Region of origin** | **Controls N** | **Cases N** | **Direction** | **OR [95% CI]** |
| --- | --- | --- | --- | --- | --- | --- | --- |
| ALS | <7/<7 vs. =>7/=>7 | 0.80 | NorthAmerica-NorthEU-SouthEU-UK | 9481 | 2039 | +-++ | 0.97 [0.75-1.25] |
|  | <7/<7 vs. 7-16/7-16 | 0.58 | NorthAmerica-NorthEU-SouthEU-UK | 9481 | 2039 | +-++ | 0.93 [0.71-1.22] |
|  | <7/<7 vs. =>7/=>17-45 | 0.15 | NorthAmerica-NorthEU-SouthEU-UK | 9481 | 2039 | +-++ | 1.77 [0.81-3.89] |
| FTD | <7/<7 vs. =>7/=>7 | 0.46 | NorthAmerica-NorthEU-SouthEU-UK | 9481 | 1016 | ++-+ | 1.11 [0.84-1.48] |
|  | <7/<7 vs. 7-16/7-16 | 0.56 | NorthAmerica-NorthEU-SouthEU-UK | 9481 | 1016 | +--+ | 1.09 [0.81-1.47] |
|  | <7/<7 vs. =>7/=>17-45 | 0.40 | NorthAmerica-NorthEU-SouthEU-UK | 9481 | 1016 | ++-+ | 1.45 [0.61-3.45] |
| FTD spectrum | <7/<7 vs. =>7/=>7 | 0.83 | NorthAmerica-NorthEU-SouthEU-UK | 9481 | 3271 | +-++ | 1.02 [0.83-1.26] |
|  | <7/<7 vs. 7-16/7-16 | 0.98 | NorthAmerica-NorthEU-SouthEU-UK | 9481 | 3271 | +-++ | 1.00 [0.81-1.25] |
|  | <7/<7 vs. =>7/=>17-45 | 0.36 | NorthAmerica-NorthEU-SouthEU-UK | 9481 | 3271 | +--+ | 1.35 [0.71-2.59] |
| PD | <7/<7 vs. =>7/=>7 | 0.42 | NorthAmerica-NorthEU-SouthEU | 1916 | 314 | +-- | 1.42 [0.61-3.32] |
|  | <7/<7 vs. 7-16/7-16 | 0.34 | NorthAmerica-NorthEU-SouthEU | 1916 | 314 | +-- | 1.55 [0.64-3.79] |
|  | <7/<7 vs. =>7/=>17-45 | 0.64 | NorthAmerica-NorthEU-SouthEU | 1916 | 314 | --- | 0.52 [0.03-8.36] |
| AD | <7/<7 vs. =>7/=>7 | 0.75 | NorthAmerica-NorthEU-SouthEU | 1916 | 868 | +-+ | 0.94 [0.66-1.35] |
|  | <7/<7 vs. 7-16/7-16 | 0.59 | NorthAmerica-NorthEU-SouthEU | 1916 | 868 | +-- | 0.90 [0.62-1.31] |
|  | <7/<7 vs. =>7/=>17-45 | 0.48 | NorthAmerica-NorthEU-SouthEU | 1916 | 868 | +-+ | 1.46 [0.52-4.15] |

**Table S5.** Results of the fixed-effects inverse variance meta-analysis.

Abbrevations: NorthAmerica = North-America, NorthEU = Northern Europe, SouthEU = Southern Europe, UK = United Kingdom. ALS = amyotrophic lateral sclerosis, FTD = frontotemporal dementia, FTD spectrum = bvFTD, primary progressive aphasia, corticobasal degeneration (CBD) and progressive supra nuclear palsy (PSP), PD = Parkinson’s disease, AD = Alzheimer’s disease. OR = odds ratio, CI = confidence interval.
