# Supplementary material for: Letter to the editor on a paper by Kaivola et al. (2020): carriership of two copies of C9orf72 hexanucleotide repeat intermediate-length alleles is not associated with amyotrophic lateral sclerosis or frontotemporal dementia
Source: Acta Neuropathol Commun. 2022 Sep 21;10:141. doi: 10.1186/s40478-022-01438-0 (PMC9494883; doi:10.1186/s40478-022-01438-0)
Supplement: Supplementary file 2 — Additional file 2. [file 40478_2022_1438_MOESM2_ESM.pdf]

Supplementary material de Boer et al., 2022.

Raw PCR data examples of samples from cohort of Reus et al., 2021

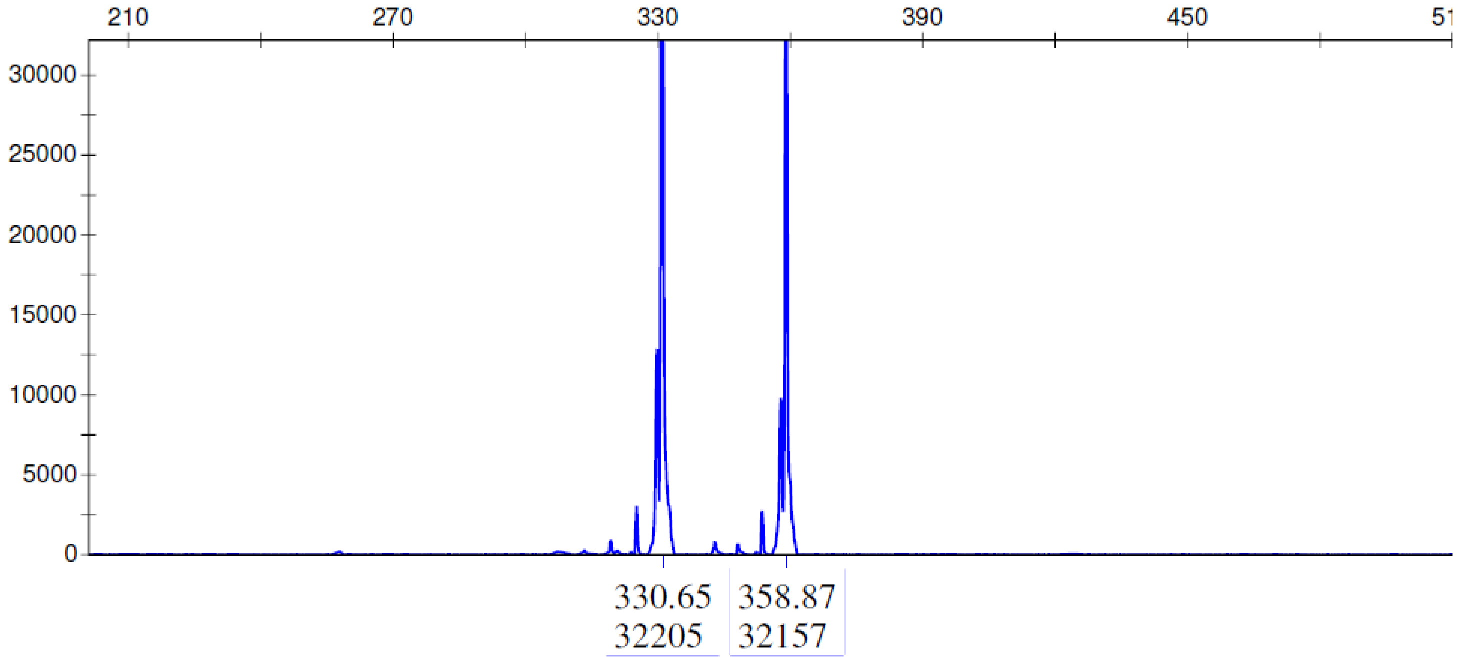

Repeat length 6 and 11

Explanation: 305 is repeat length of 2

330-305=25/6=4 repeat difference + 2 original = 6
358-305=53/6=9 repeat difference + 2 original = 11

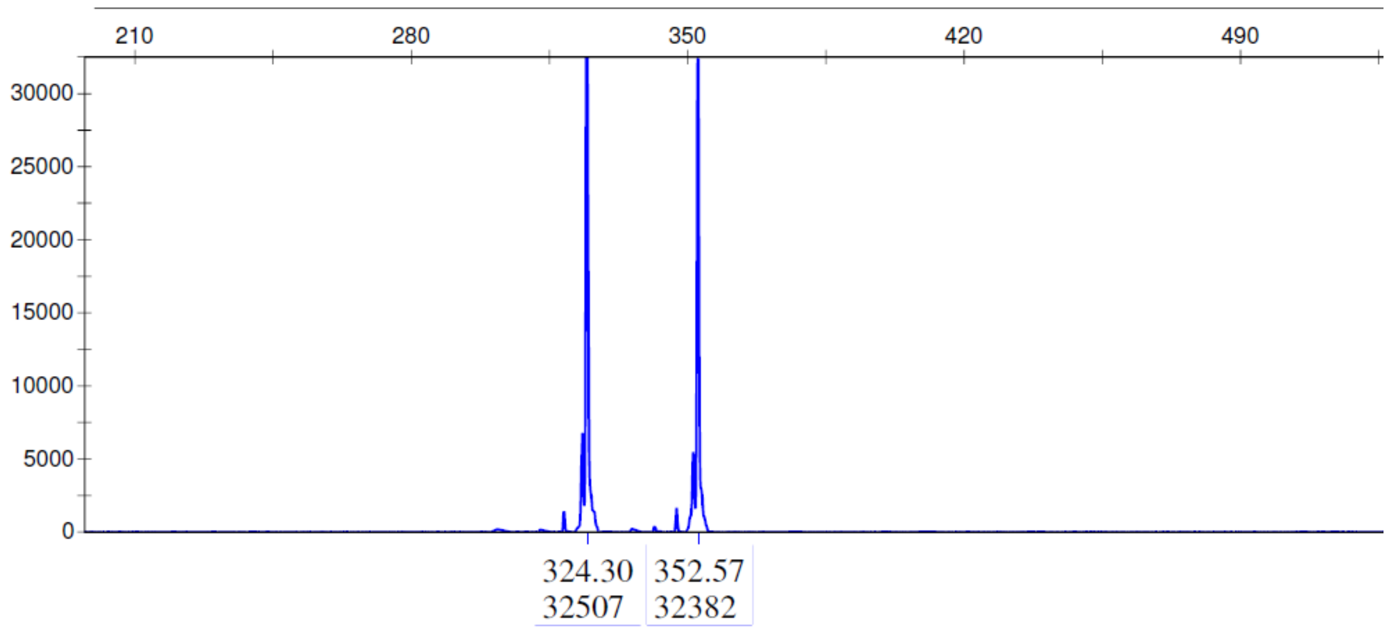

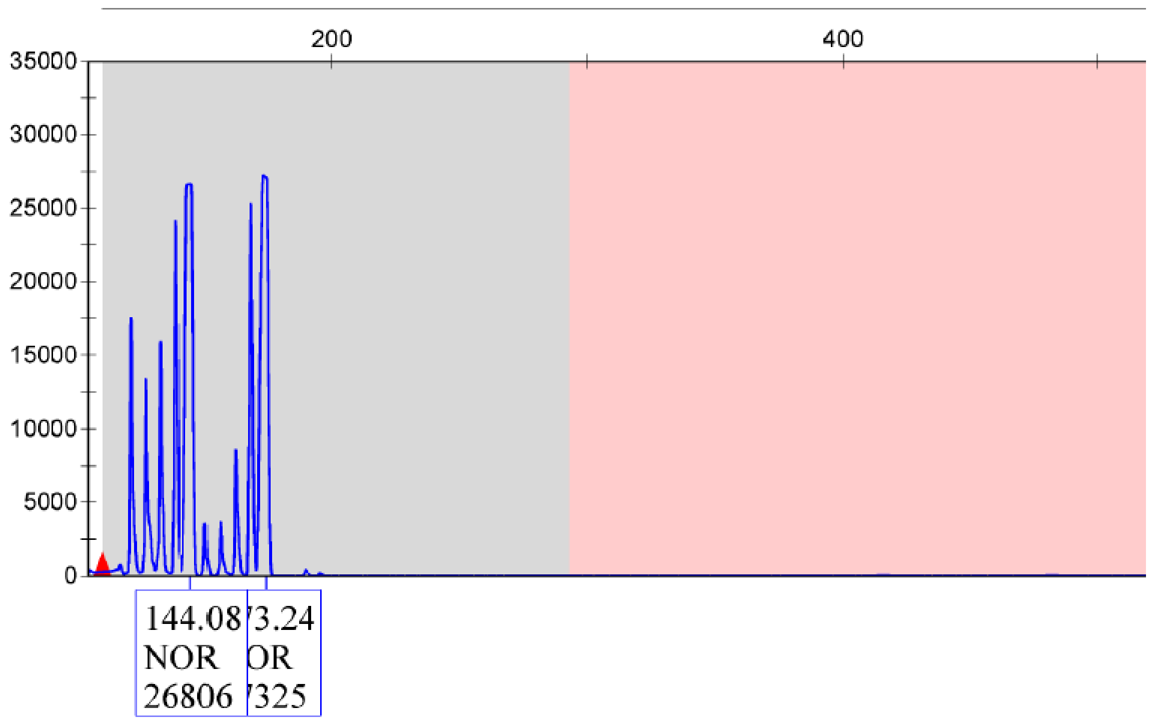

Repeat length 5 and 10, confirmed with Asuragen

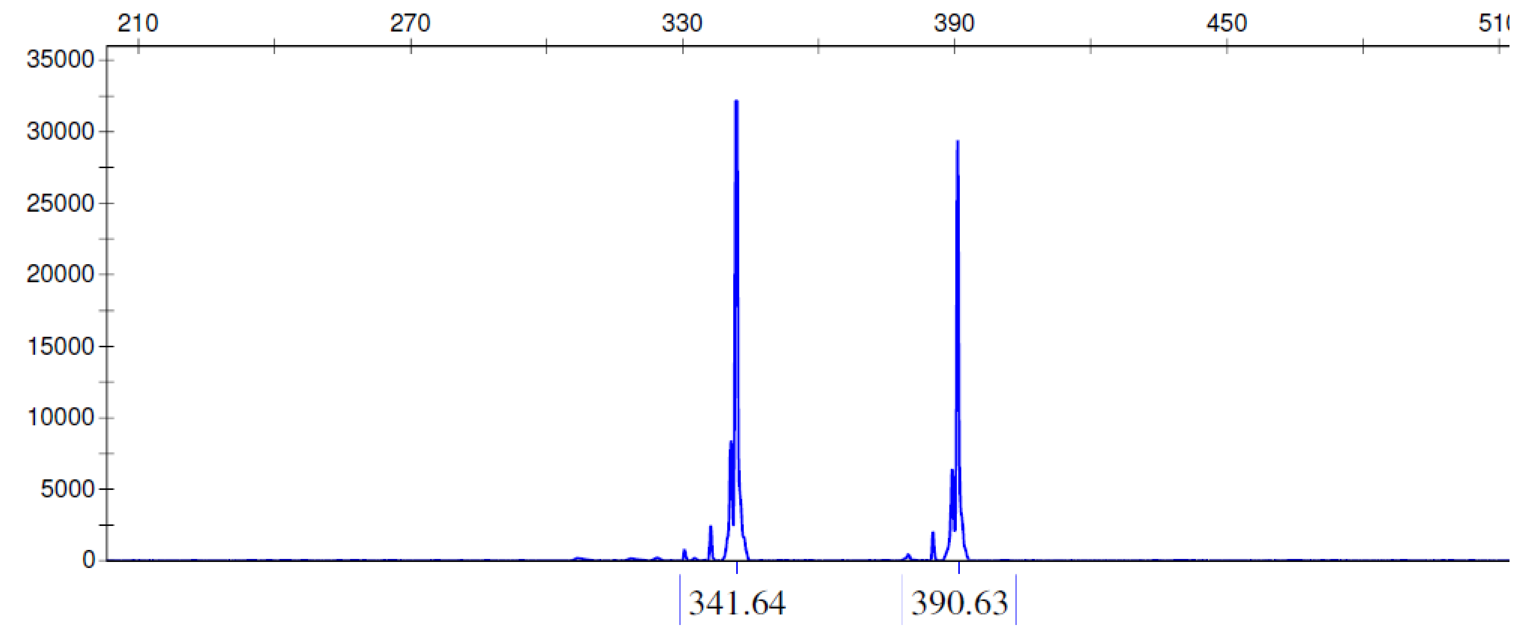

Repeat length 8 and 16

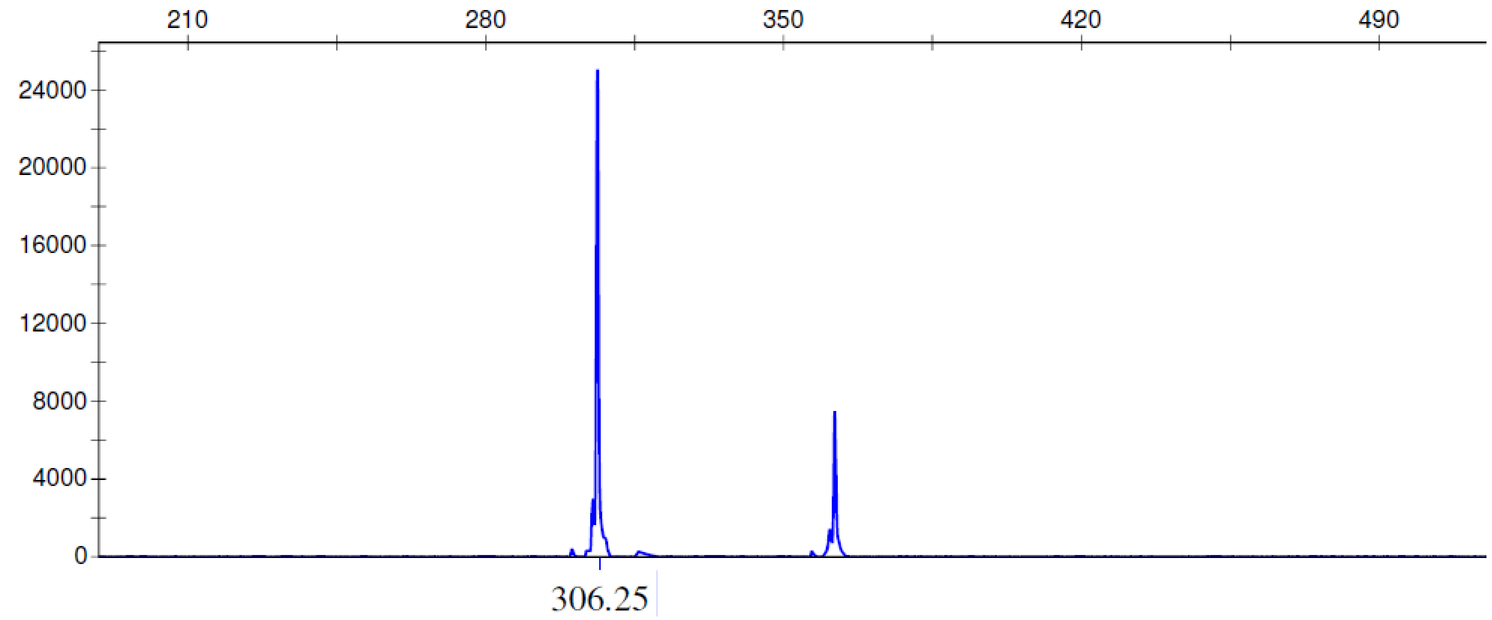

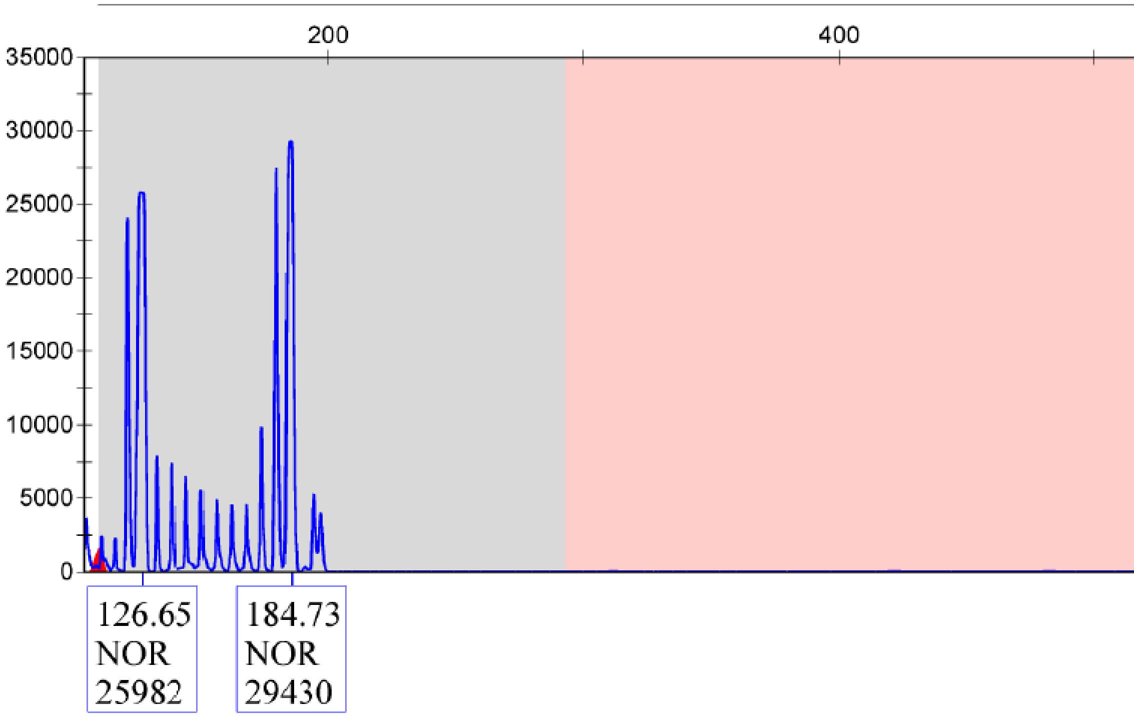

Repeat length 2 and 12, confirmed with Asuragen

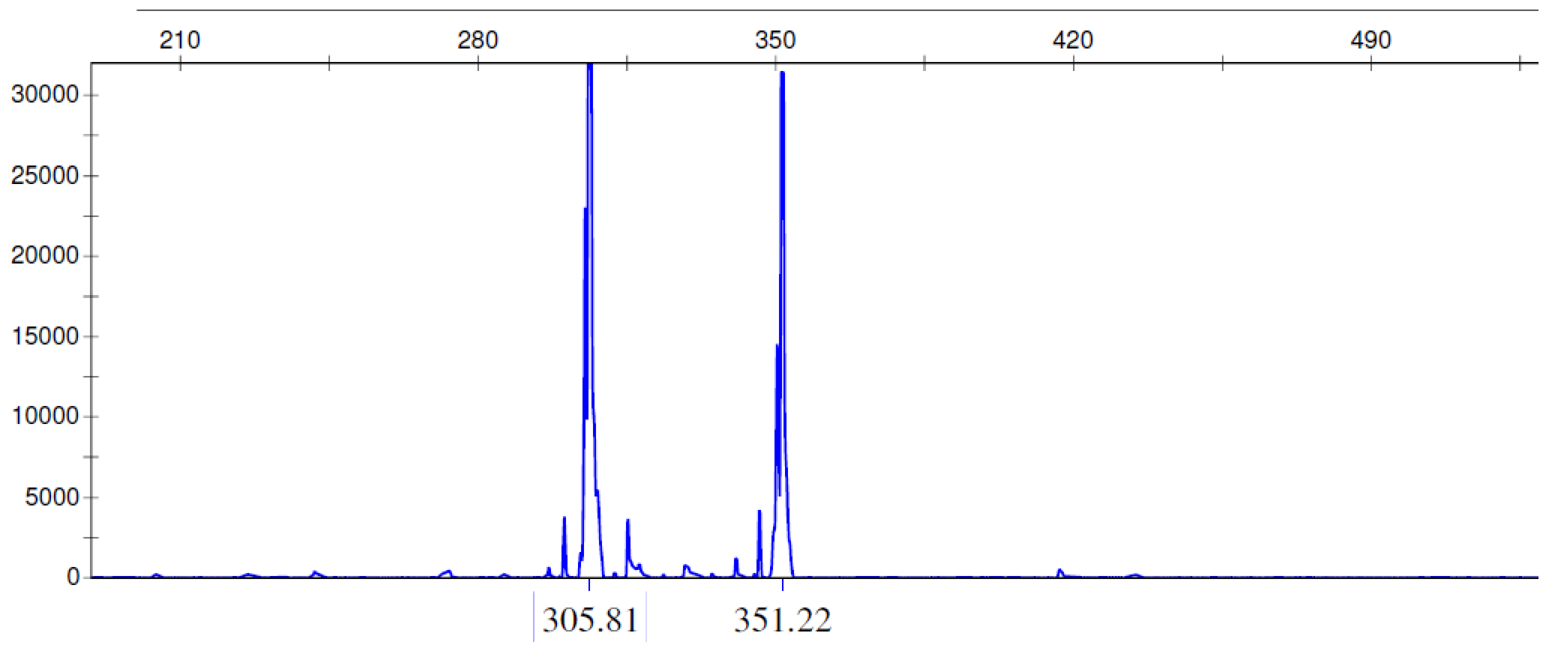

Repeat length 2 and 10

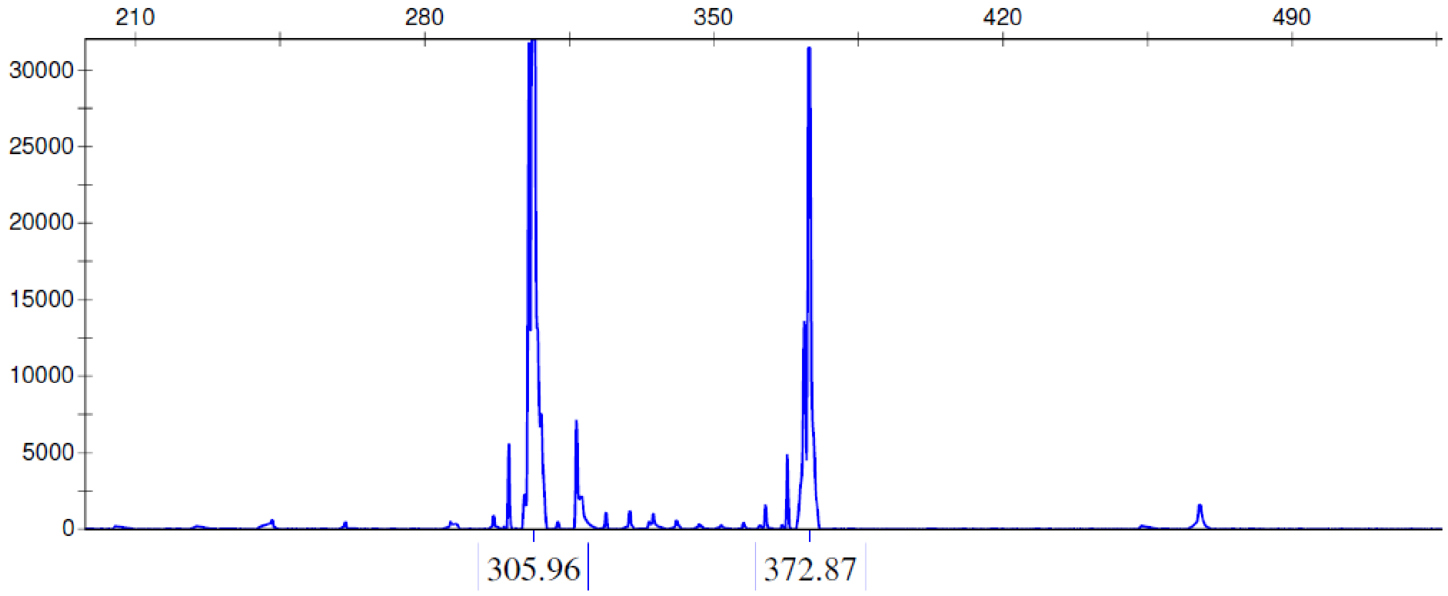

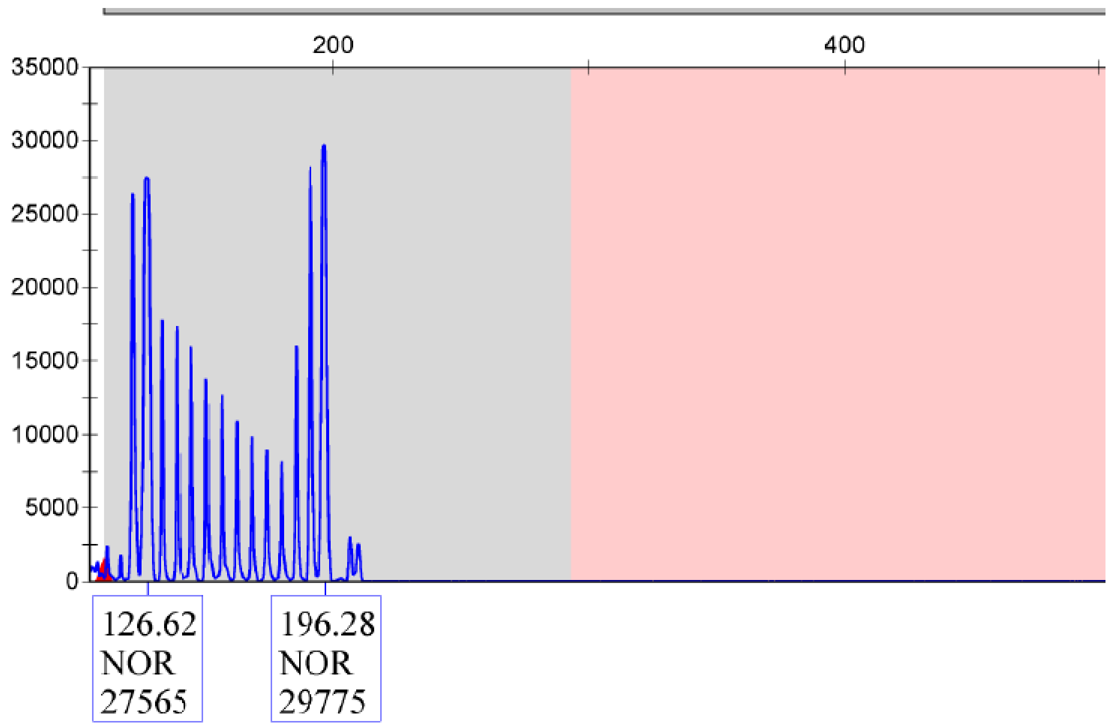

Repeat length 2 and 13, confirmed with Asuragen

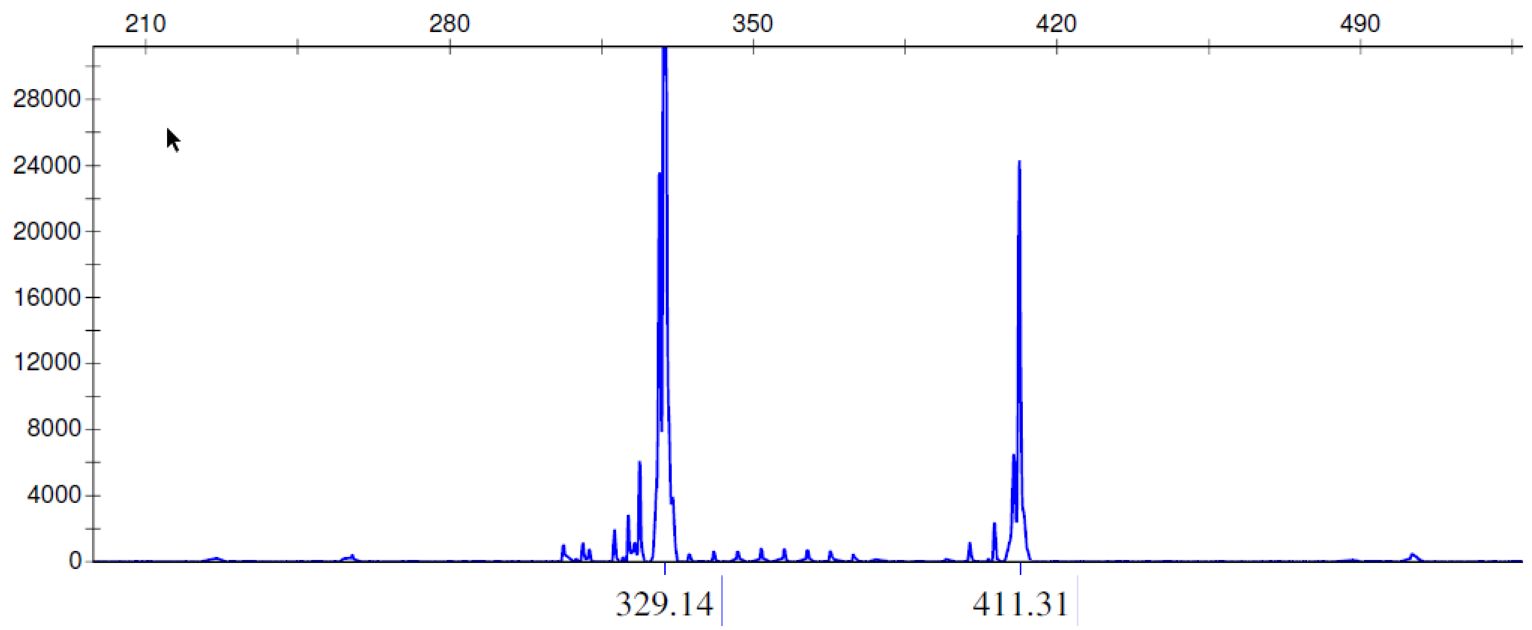

Repeat length 6 and 20

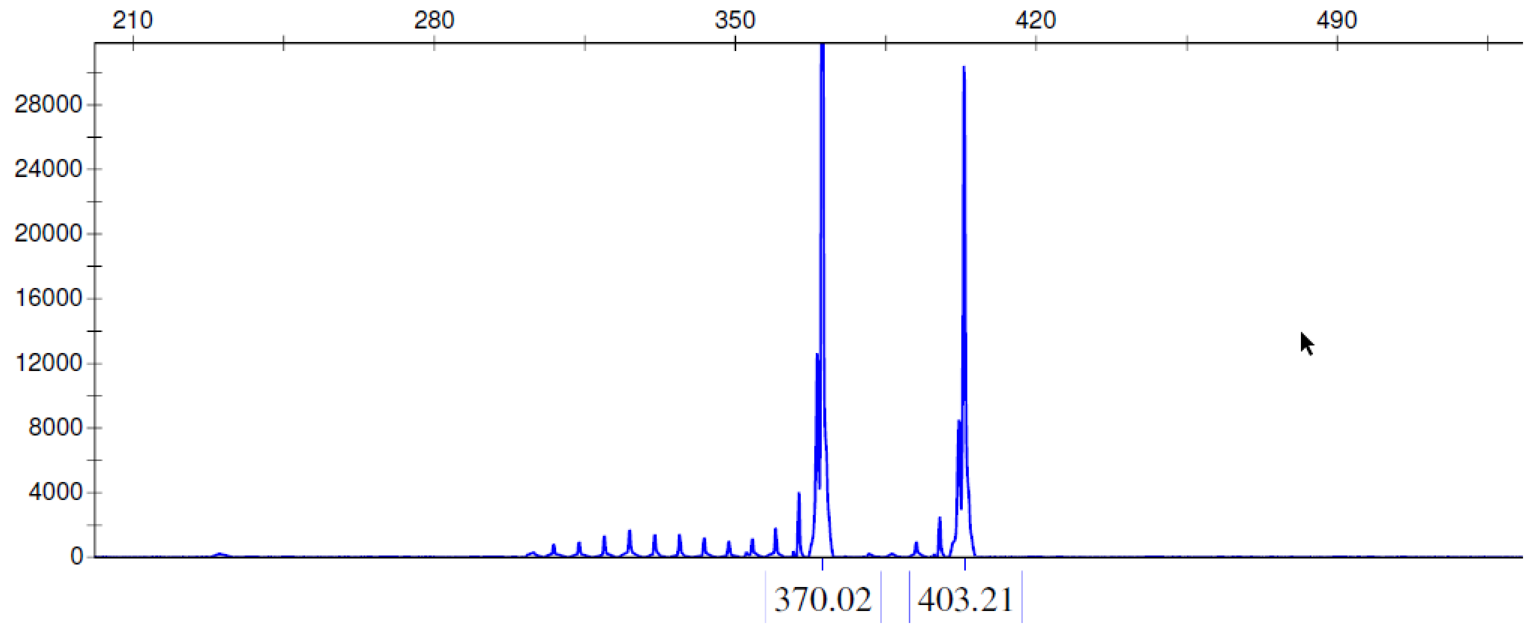

Repeat length 13 and 18

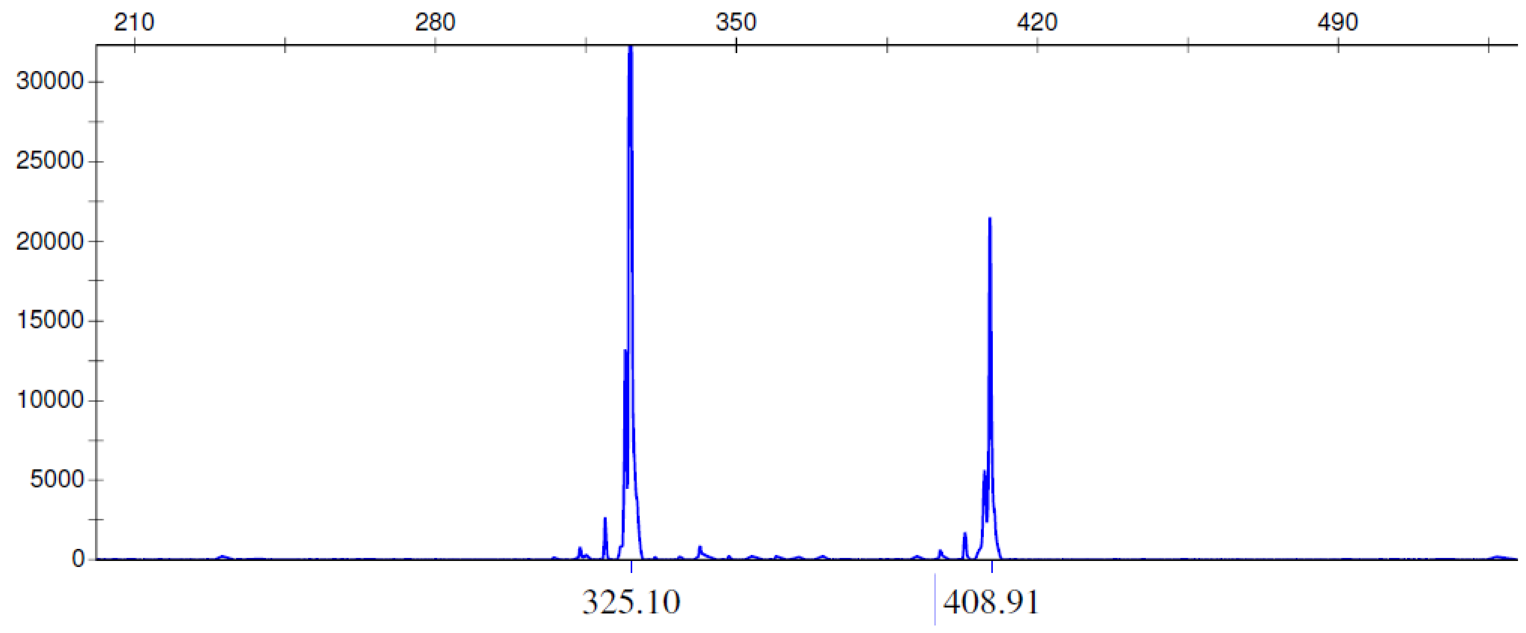

Repeat length 5 and 19

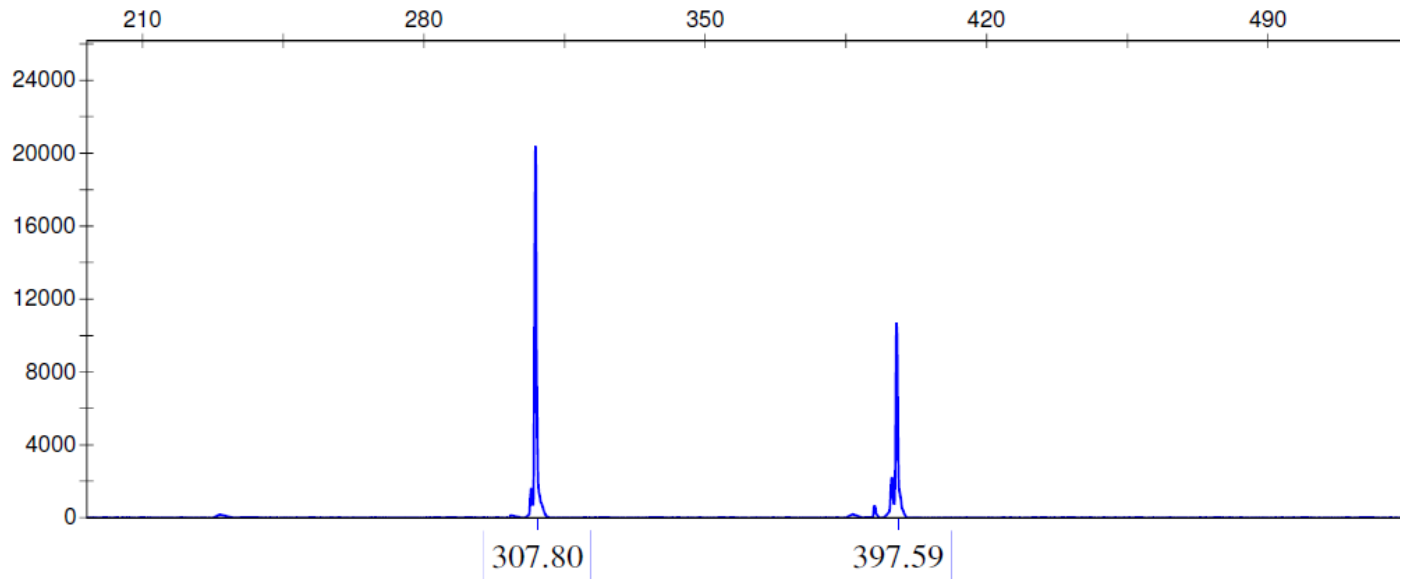

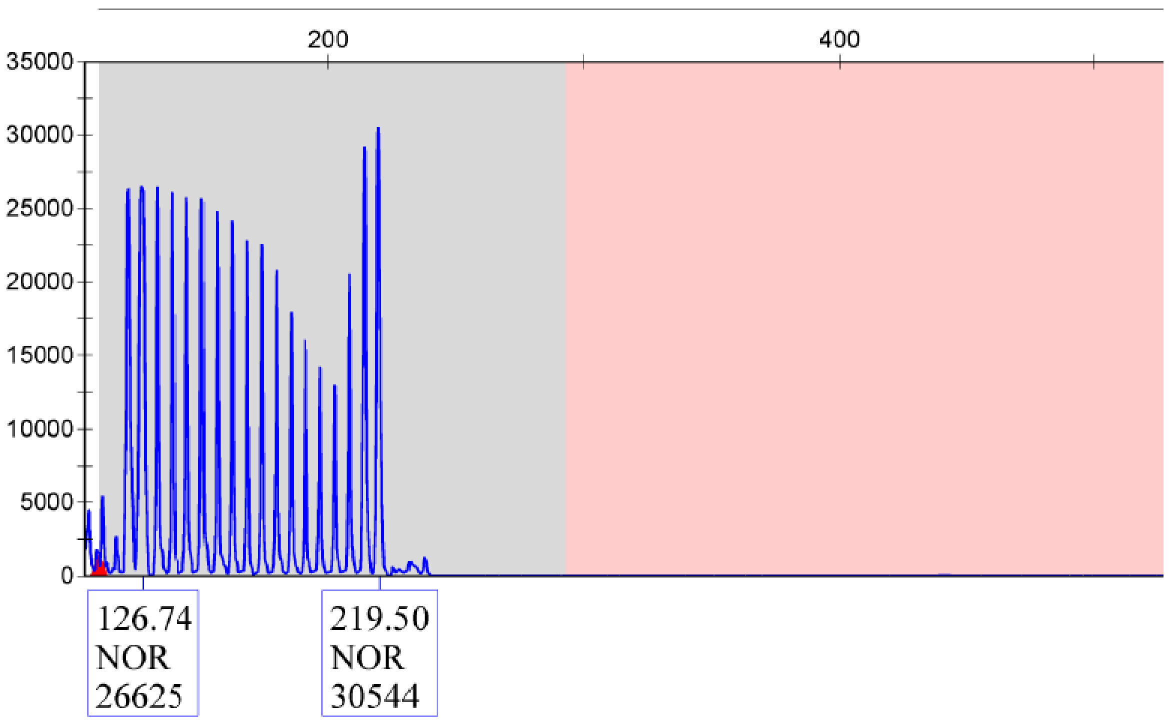

Repeat length 2 and 17, confirmed by Asuragen

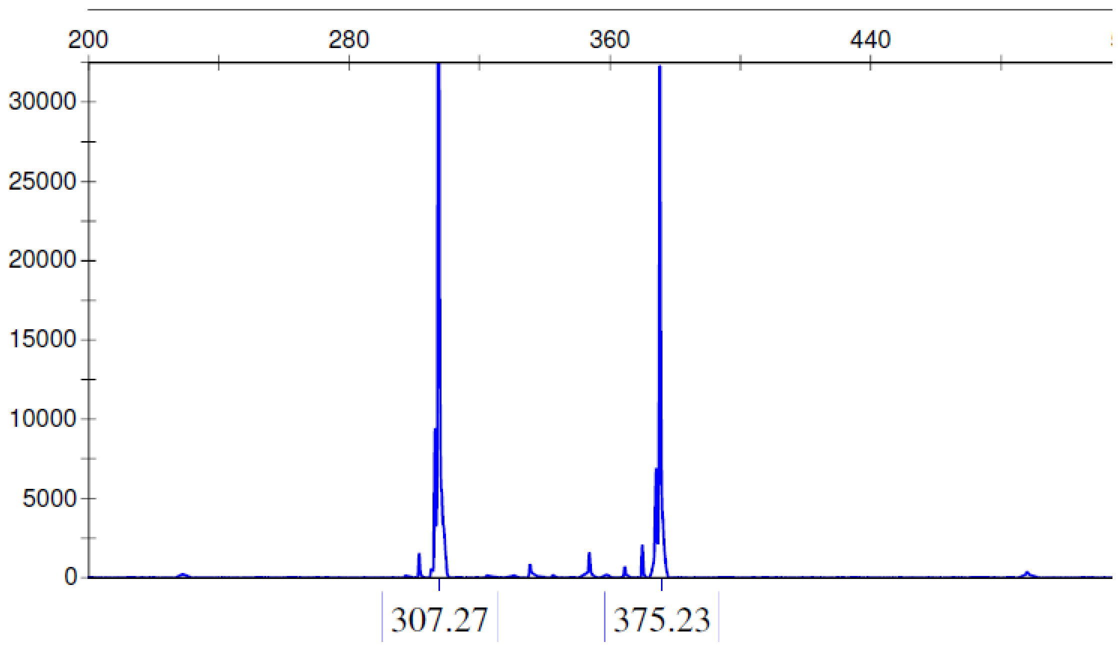

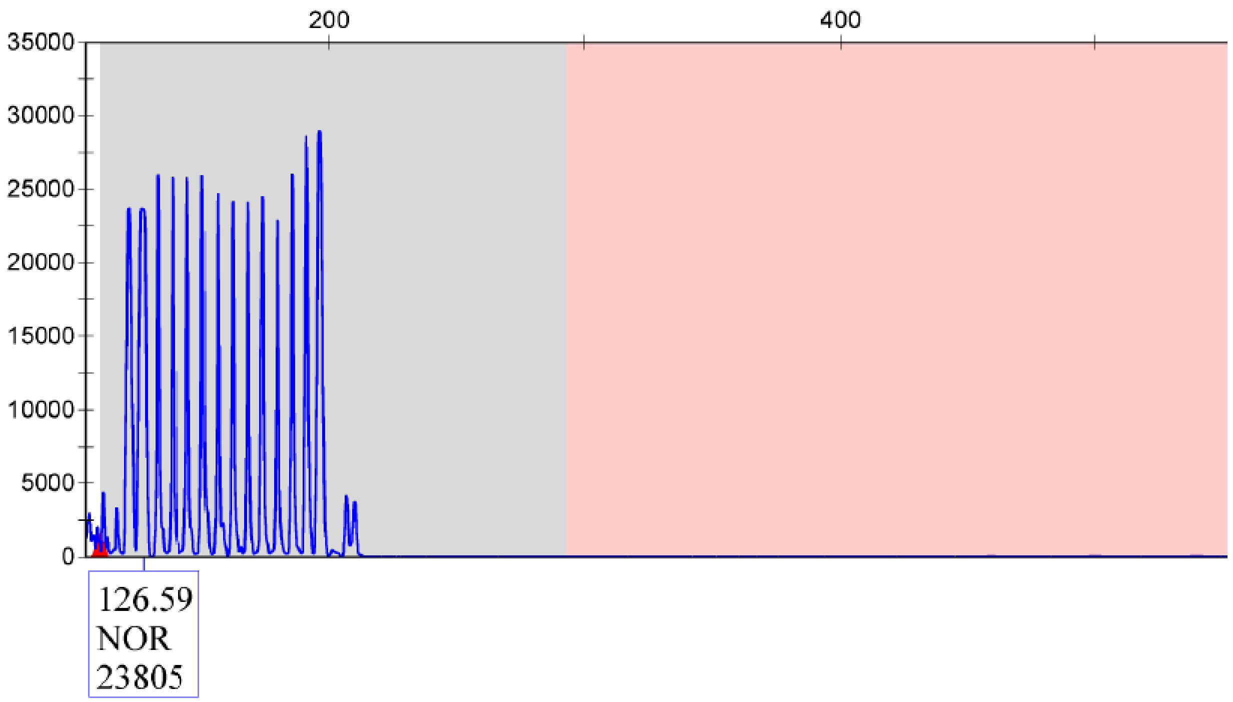

Repeat length 2 and 14, confirmed by Asuragen
